# Consuming treats with amino acids, osmolytes and sodium increased total water intake and decreased urine specific gravity of healthy cats

**DOI:** 10.1177/1098612X261477044

**Published:** 2026-07-30

**Authors:** Pauline AL Kosmal, Anna K Shoveller, Sydney Banton, Kira Cheer, Nicolas Farentino, Lawrence E Armstrong

**Affiliations:** 1 Department of Animal Biosciences, University of Guelph, ON, Canada; 2 Korey Stringer Institute, University of Connecticut, Storrs, CT, USA

**Keywords:** Amino acids, electrolytes, hydration, osmolarity, salt, specific gravity, water intake

## Abstract

**Objectives:**

It is estimated that cats do not drink enough water, particularly those maintained on a dry diet. As such, the objective of this study was to determine the effects of providing two different high-moisture lickable treats containing glycine, alanine, taurine and betaine, and two sodium levels, on water intake and hydration indices in cats fed dry diets.

**Methods:**

A total of 12 adult cats were provided treatments in a 3 × 3 replicated Latin square design for 5 days per leg: dry diet (CON), CON + one treat with 0.99% sodium (TRT A) and CON + one treat with 1.55% sodium (TRT B). Daily free water intake (FWI) was measured using fountains, and total water intake (TWI) was calculated as FWI + moisture from food and treats. Urine specific gravity (USG) and pH were measured daily; urine protein (UP), urine creatinine (CR) and the protein:creatinine (UP:CR) ratio were assessed on the final day of each leg. Fasted blood samples were collected for serum biochemistry.

**Results:**

Daily TWI was lower on CON than on TRT B (*P* = 0.001) and TRT A (*P* = 0.009). Average USG was higher on CON than on TRT B (*P* <0.001) and TRT A (*P* = 0.005). UP and CR were also higher on CON (*P* <0.001; *P* = 0.003). Daily TWI, average USG, UP and CR were similar on TRT A and TRT B. Urine pH varied by day (*P* <0.001), but treatments did not differ. Magnesium was greater in CON than TRT B (*P* = 0.006). Average FWI, UP:CR ratio and all other biochemistry values did not differ.

**Conclusions and relevance:**

These data support that feeding either TRT A or TRT B increased the TWI of domestic cats fed a dry diet, and additional benefits accrued in lower USG, UP and CR.

## Plain language summary

Consuming high-moisture lickable treats increased total water intake and diluted the urine of healthy cats

Some indoor domestic cats fed only dry food may not consume enough water, and methods to increase their water intake should be explored. Here, we tested the impact of feeding a newly developed hydration treat on water intake and hydration markers in cats. For this, 12 neutered adult male cats were fed one of three treatments for 5 days each: dry diet (CON), dry diet + one treat with 0.99% added sodium (TRT A) and dry diet + one treat with 1.55% added sodium (TRT B). Body weight and feed intake were monitored and kept constant throughout the study. Free water intake was recorded using an app-based water fountain, and total water intake was estimated as free water intake + the water from food and treats. Urine was collected daily to measure urine concentration, pH and waste products. Blood samples were also collected to further assess hydration and health. Total water intake was greater, and urine concentration and waste products were lower, when the cats were fed either treat. Overall, feeding either treat increased total water intake but not free water intake. However, the hydration state was improved in these cats.

## Introduction

It is estimated that domestic cats do not drink enough water, given the low moisture content of commercial dry diets (3–11% moisture) relative to the natural diet of feral cats (65–75% moisture).^1^ Previous research reports that healthy adult cats fed a wet (>65% moisture) or dry diet (<10% moisture) consumed an average of 42.0–52.0 ml/day and 27.0–41.0 ml/kg body weight (BW) of total water per day, respectively.^2,3^ Importantly, most of these values fall below the current National Research Council recommendation of 50–60 ml/kg BW daily,^4^ especially for cats fed commercial dry diets. However, this recommendation is based on indispensable losses in a euhydrated cat or dog that is urinating normally^5^ and does not account for differences in diet, environment or activity levels.

Water balance is tightly regulated; however, biological indicators of hydration state can be influenced by various factors. For example, blood urea nitrogen (BUN) and the urine protein (UP):creatinine (CR) (UP:CR) ratio are used to assess renal function in animals,^6^ but can be affected by protein intake.^7^ In cats, urine specific gravity (USG), UP, CR, urine pH, osmolarity (mOsm/l) or osmolality (mOsm/kg), volume (or output), calcium oxalate (CaOx), struvite and/or serum biochemistry, and a complete blood count (CBC) have been used to assess the impact of diet and water format on physiological biomarkers of hydration or the risk of urine crystal formation.^2,3,8–26^ In colony cats, previous research has reported an inverse relationship between urinary mOsm/kg and total water intake (TWI), a positive relationship between urinary mOsm/kg and USG, and no relationship between serum mOsm/kg and urinary mOsm/kg.^20^ Similar results were found in a study assessing the effect of water viscosity (by adding 1% methylcellulose to free water) on water intake in healthy adult cats, in which mOsm/kg was not affected after 28 and 56 days of treatment, despite increases in TWI and decreases in USG.^25^

In humans, plasma or serum mOsm/l or mOsm/kg can be used to evaluate hypertonic dehydration, in which a high mOsm/l or mOsm/kg value indicates greater solute concentration (in 1 l or 1 kg of solute, respectively^27,28^) and less intracellular water. Thus, both mOsm/l and mOsm/kg can help to assess underhydration.^29,30^ Alternatively, USG can provide information on the renal ability to concentrate urine.^31^ USG is considered valid for dynamic hydration assessments in humans under controlled laboratory conditions^29^ and has previously been used to assess the effects of dietary treatments, such as nutrient-enriched water (NW),^20,21^ dietary moisture content,^2,3,15,17^ and salt content^12,23^ in healthy cats. Higher USG and mOsm, along with lower urine volume, often indicate higher plasma arginine vasopressin concentrations and can be used to distinguish low- and high-water drinkers and to assess disease risk.^32^ In healthy, euhydrated (well-hydrated) men and women, USG is in the range of 1.012–1.026,^33,34^ whereas the majority of apparently healthy cats have a USG above 1.035.^35^ However, there is no consensus on the normal USG range in cats, and this does not account for cats fed high-moisture diets. Although cats can concentrate their urine more than humans and most other mammals,^36^ a low water intake and a high USG can also indicate underhydration. Cats fed an exclusively dry commercial diet had a 2.64 times greater risk of developing feline lower urinary tract disease (FLUTD) than cats fed a mixed diet (canned and dry).^37^ In addition, there is an established association between water consumption and chronic kidney disease (CKD)^38,39^ and kidney stones^40^ in humans. Given the prevalence of CKD (up to 50% in a randomly selected feline population admitted to a single veterinary practice^41^) and urolithiasis (up to 21% of diagnoses in cats with FLUTD^42^), it is suspected that domestic indoor cats, especially those that consume low-moisture diets, may benefit from a greater water intake to support urinary and renal health.

Notably, many studies report that feeding canned diets increases water intake and, consequently, decreases USG.^2,3,12,15,17^ Despite these benefits, feeding wet food may not be feasible for all cat owners. Surveys of American and Australian^43^ and UK^44,45^ cat owners reported that 18–45% feed their cats a solely commercial dry diet, compared with <10% who feed a solely canned (wet) diet. Owners may feed dry food owing to cost, perceived convenience, ease of storage and feeding, and compatibility with multi-cat households. Previous research suggests that providing a nutrient complex may increase water intake in cats fed dry diets. A greater TWI and lower USG, but higher mOsm/l, were reported in cats supplemented with NW containing added whey protein and glycerin, compared with those provided plain tap water.^20,21^ Other attempts to increase TWI include supplying water fountains or circulating water. Although some individual cats showed a preference for a water system (free-falling, still or circulating), average daily water intake did not differ between treatment groups across a 14-day measurement period.^22^ Thus, these methods may not be effective for all cats.

Dietary interventions to increase water intake in cats are of interest. Providing a nutrient complex in a supplement or treat is a practical, potentially effective strategy. Ingredients such as glycine, alanine, taurine and betaine are of interest because of their potential to improve intestinal water absorption. Therefore, the objectives of this study were to determine the effects of two novel hydration treats containing glycine, alanine, taurine and betaine, and two sodium (Na) levels, on water intake and physiological indices of hydration state in healthy adult cats in a controlled environment. We hypothesized that both treats would increase free water intake (FWI) and TWI in healthy neutered adult male cats and decrease USG, UP, CR, BUN and calculated mOsm/l compared with cats fed only a dry diet.

## Materials and methods

### Animals and housing

All procedures involving animals were approved by the University of Guelph Animal Care Committee in compliance with the Canadian Council on Animal Care and the provincial Animals in Research Act. A total of 12 neutered male domestic shorthair colony cats, aged 5–6 years, with a mean (± SD) BW of 4.51 ± 0.43 kg and body condition score (BCS) of 4–6/9, were used in this study. The cats were group housed in an indoor, enriched, free-living environment (7.1 m × 5.8 m) with a 12-h light-dark cycle. This study was conducted in August 2025.

### Diet and study design

This study employed a replicated 3 × 3 Latin square design with four replicates per group. Cats were transitioned from a commercial wet diet (Acana Premium Paté, Beef Recipe; Champion Petfoods) to a dry complete diet (Table 1) 8 days before the start of the study. Cats were randomly divided into three groups and were fed one of three dietary treatments: control diet (CON), control diet + one high moisture lickable treat with 0.99% Na (TRT A) or control diet + one high moisture lickable treat with 1.55% Na (TRT B) for 5 days each. At the time of feeding, cats were individually crated and fed 100% of the diet at 0700 h, and those on a treatment received a 17 g high-moisture treat at 1600 h (Peturio, doing business as Cat Years) (Table 2). Cats were given up to 1 h to consume the complete diet and 30 mins to consume the treat. These cats have been previously acclimated to once-a-day feeding within a 1-h window.^46–48^ Weekly BW was recorded using a digital scale before feeding, and BCS was assessed on a 9-point scale.^49^ Cats were fed to maintain BW, based on historical individual intake data, and to meet their maintenance energy requirements. The individual cats received the same energy (caloric) content across treatment periods.

**Table 1:** The guaranteed analysis and sodium content for the dry complete diet fed to adult cats

Guaranteed analysis (%)	
Crude protein (min)	40.0%
Crude fat (min)	15.0%
Crude fiber (max)	3.0%
Ash (max)	8.0%
Sodium (max)	0.43%
Moisture (max)	6.0%
ME^a^ (kcal/kg)	3735

Values expressed on an as-fed basis

a Metabolizable energy (ME) calculated according to the Nutrient Requirements of Dogs and Cats.^4^ The diet complies with guidelines from the Association of American Feed Control Officials for adult cats at maintenance. Ingredients: chicken meal, whole red and green lentils, whole yellow and green peas, dehydrated chicken, pea starch, fresh chicken and turkey giblets, turkey, herring meal, turkey meal, sunflower oil, fresh chicken, egg powder, miscanthus grass, pollock oil, fresh whole egg, spray dried sardine, potassium chloride, vitamin E, salt, dried kelp, choline chloride (choline), zinc proteinate, taurine, biotin premix, natural mixed tocopherols, vitamin B premix, copper proteinate

**Table 2:** Guaranteed analysis and sodium content of two novel hydration treats fed to 12 healthy adult male cats

Guaranteed analysis (%)	Treat	
	TRT A	TRT B
Crude protein (min)	14.0	14.0
Crude fat (min)	0.4	0.4
Crude fiber (max)	0.1	0.1
Moisture (max)	85.0	85.0
Sodium (max)	0.99	1.55

Values expressed on a dry matter basis, excluding % moisture (as-fed)

Ingredients: tuna broth, wild caught tuna, chicken liver, glycine, L-alanine, purified mineral salt, taurine, betaine, natural tapioca starch, natural guar gum, natural dried yeast

TRT A = dry diet + one treat with 0.99% sodium; TRT B = dry diet + one treat with 1.55% sodium

### Water intake

Average daily FWI was defined as the amount of water the cat electively consumed. Estimated TWI included FWI, the maximum moisture content of the diet (6.1%) and the treat (85.0%) and excluded metabolic water. Individual FWI was recorded using seven Felaqua connect water fountains (Sure Pet; Merck). The water fountains replaced their usual water bowls, starting 3 months before the research trial began. The research team validated the fountains before use, as described in van Diggelen et al.^50^ The water fountains were cleaned and refilled daily with distilled water. Data were collected from the water fountains using a radio-frequency identification tag attached to a harness, transmitted to the PetSure app (SureFlap) and then transferred to an encrypted, password-protected laptop for analysis. When multiple cats drank simultaneously, each drinking event was recorded as the maximum time and total volume consumed. Although duration was not adjusted, the volume consumed during each group’s drinking event was divided by the number of cats present.

### Urine collection and analysis

An observer familiar with the cats collected free-catch urine samples during two daily windows: once between 0800 h and 1600 h, and once between 1630 h and 1900 h. When a cat approached the litter box, the observer positioned a sterile collection cup to capture the midstream sample. The cats were previously acclimated to this method, and free-catch midstream urine samples are considered effective in habituated cats.^51^ Urine samples were refrigerated at 4°C until analysis. USG and pH were measured within 4 h of collection. The pH of each sample was measured using an AE150 benchtop pH meter (Fisher Scientific) and a 13-620-AE6 probe (Fisher Scientific). USG was measured using an ATC portable refractometer (Agriculture Solutions). Measurements of pH and USG were taken in duplicate, and the mean was used for analysis. The values were reported for each day and averaged across the 5-day treatment periods to improve the accuracy of USG as an indicator of dynamic hydration. On the last day of each leg (day 5), after pH and USG measurements were taken, 5–10 ml of urine was aliquoted and sent to the Animal Health Laboratory (AHL; Laboratory Services Division, University of Guelph, ON) for UP and CR analysis using photometric testing methods for spot urine collection.

### Blood collection and analysis

On the last day of each leg, a 1.5–2.0 ml blood sample was collected in nine cats (n = 3 per treatment group) via saphenous venipuncture with a serum vacutainer system (Becton, Dickinson and Company). The same cats were used at all sampling points, except for one, which was excluded because they could not be safely restrained for blood collection using Fear Free handling^52^ techniques at the last sampling point. After a 30-min clotting period, serum was separated by centrifugation at 2500 × *g* for 15 mins at 4°C. Fresh serum samples were tested at AHL for blood biochemistry using photometric testing methods. Osmolarity was calculated as Osmc (mmol/l) = 1.86 ([Na] + [K]) + [glucose] + [urea]. AHL’s reference intervals for biochemistry were based on 40 fasted, clinically healthy, adult cats of various breeds.

### Statistical analyses

Descriptive statistics were used to calculate the mean concentration ± SD for all variables. Statistical analyses were performed using Statistical Analysis System (version 9.4; SAS Institute). The PROC GLIMMIX procedure was used to assess BW and dry matter intake, with treatment included as a fixed effect and individual cat and treatment leg as random effects (see Table S1 in the supplementary material). The PROC MIXED procedure for repeated-measures data, with treatment, day and their interaction as fixed effects and individual cats and leg as random effects, was used to analyze FWI, TWI, USG and pH. The PROC MIXED procedure was also used to assess BUN, UP, CR, UP:CR ratio and serum biochemistry parameters, with treatment as the fixed effect and individual cat and treatment leg as the random effects.

Tukey post-hoc least-squares means comparisons were performed when significant differences were observed. Given that TWI and USG were significantly affected by treatment consumption, an additional post-hoc PROC MIXED analysis with the Satterthwaite approximation for degrees of freedom was conducted to assess this relationship across treatments, using data from individual cats. Significance was considered at *P* ⩽0.05 for all comparisons.

## Results

### Water intake

Daily FWI was not affected by treatment (*P* = 0.230), day (*P* = 0.967) or their interaction (*P* = 0.911). The estimated average daily TWI was affected by treatment (*P* <0.001), but not the day (*P* = 0.959) or their interaction (*P* = 0.922) (Table 3). The estimated daily TWI of cats in the CON group was lower than on TRT B (*P* = 0.001) and TRT A (*P* = 0.009). The estimated daily TWI of cats on TRT A and TRT B was similar (*P* = 0.188).

**Table 3:** Daily free and total water intake of 12 healthy adult male neutered cats fed a dry diet (CON,) CON + one treat with 0.99% sodium (TRT A) or CON + one treat with 1.55% sodium (TRT B) for 5 days

Variable		Treatment			*P* value		
	Day	CON (n = 12/day)	TRT A (n = 12/day)	TRT B (n = 12/day)	Trmt	Day	Trmt × day
FWI (ml)	1	80 ± 19	85 ± 23	100 ± 55	0.230	0.967	0.911
	2	87 ± 28	84 ± 28	100 ± 42			
	3	84 ± 22	98 ± 33	97 ± 40			
	4	91 ± 24	90 ± 28	99 ± 25			
	5	97 ± 37	90 ± 31	90 ± 14			
	Ave	88 ± 27	89 ± 29	97 ± 38			
TWI (ml)	1	83 ± 12	103 ± 25	119 ± 52	<0.001	0.959	0.922
	2	90 ± 26	102 ± 42	118 ± 57			
	3	87 ± 23	116 ± 31	116 ± 33			
	4	94 ± 29	108 ± 26	117 ± 32			
	5	100 ± 31	109 ± 37	109 ± 34			
	Ave	91 ± 25^b^	107 ± 32^a^	116 ± 42^a^			

Data are mean ± SD. Values with different letters are significantly different at *P* ⩽0.05

Ave = average across 5 days; FWI = free water intake; Trmt = treatment; TWI = total water intake

### Urinary parameters

A total of 149 day (pre-treat; 0800 h to 1600 h) and 45 evening (post-treat; 1630 h to 1900 h) urine samples were collected across all days and cats. Given the low number of post-treat samples collected, only the day samples were used for statistical analysis. USG of cats was affected by the treatment group (*P* <0.001), but not the day (*P* = 0.945) or their interaction (*P* = 0.773). The average USG was lower in the TRT B group than in the CON group (*P* <0.001), but TRT B was similar to TRT A (*P* = 0.445). The average USG was also lower on TRT A than on CON (*P* = 0.005). Urine pH differed by the day (*P* <0.001), but not the treatment (*P* = 0.356) or their interaction (*P* = 0.849). Urine pH was lower on day 5 than on all other days across all treatment groups (*P* <0.05 for all). No additional effects on urine pH were found (*P* >0.05) (Table 4). The UP and CR of cats were affected by the treatment (*P* <0.001; *P* = 0.003) and were lower on TRT B and TRT A than in the CON group (*P* <0.05 for both). The UP:CR ratio was not affected by treatment (*P* = 0.598) (Table 5).

**Table 4:** Urine specific gravity (USG) and pH of 12 healthy adult male neutered cats fed a dry diet (CON), CON + one treat with 0.99% sodium (TRT A) or CON + one treat with 1.55% sodium (TRT B) for 5 days

Variable		Treatment			*P* value		
	Day	CON	TRT A	TRT B	Trmt	Day	Trmt × day
USG	1	1.046 ± 0.011	1.042 ± 0.009	1.040 ± 0.008	0.009	0.944	0.773
		9	8	9			
	2	1.045 ± 0.010	1.044 ± 0.009	1.042 ± 0.007			
		10	8	10			
	3	1.044 ± 0.006	1.043 ± 0.009	1.041 ± 0.010			
		8	11	9			
	4	1.048 ± 0.006	1.042 ± 0.008	1.041 ± 0.008			
		9	11	11			
	5	1.045 ± 0.006	1.043 ± 0.010	1.040 ± 0.009			
		12	12	12			
	Ave	1.045 ± 0.006^a^	1.043 ± 0.009^b^	1.042 ±0.008^b^			
		48	50	51			
pH	1^a^	7.95 ± 0.32	8.03 ± 0.60	7.87 ± 0.56	0.356	<0.001	0.849
		9	8	9			
	2^a^	7.87 ± 0.60	7.85 ± 0.52	7.80 ± 0.56			
		10	8	10			
	3^a^	8.20 ± 0.23	7.74 ± 0.67	7.83 ± 0.63			
		8	11	9			
	4^a^	7.90 ± 0.68	7.96 ± 0.58	7.84 ± 0.64			
		9	11	11			
	5^b^	7.51 ± 0.58	7.23 ± 0.87	7.36 ± 0.72			
		12	12	12			
	Ave	7.84 ± 0.57	7.73 ± 0.71	7.72 ± 0.66			
		48	50	51			

Data are n (total number of samples collected) or mean ± SD. Values with different letters are significantly different at *P* ⩽0.05

Ave = average across 5 days; Trmt = treatment

**Table 5:** Urine protein (UP), creatinine (CR) and urine protein:creatinine (UP:CR) ratio of 12 healthy adult male neutered cats fed a dry diet (CON), CON + one treat with 0.99% sodium (TRT A) or CON + one treat with 1.55% sodium (TRT B) for 5 days

Variable	Treatment			*P* value
	CON (n = 12)	TRT A (n = 12)	TRT B (n = 12)	
UP (g/l)	0.178 ± 0.043^a^	0.163 ± 0.039^b^	0.164 ± 0.033^b^	<0.001
CR (mmol/l)	27.48 ± 9.37^a^	24.33 ± 7.43^b^	23.85 ± 7.78^b^	0.003
UP:CR ratio	0.067 ± 0.062	0.058 ± 0.051	0.075 ± 0.065	0.598

Data are mean ± SD. Values within the same row with different letters are significantly different at *P* ⩽0.05

### Change in USG and TWI

**Figure 1:**
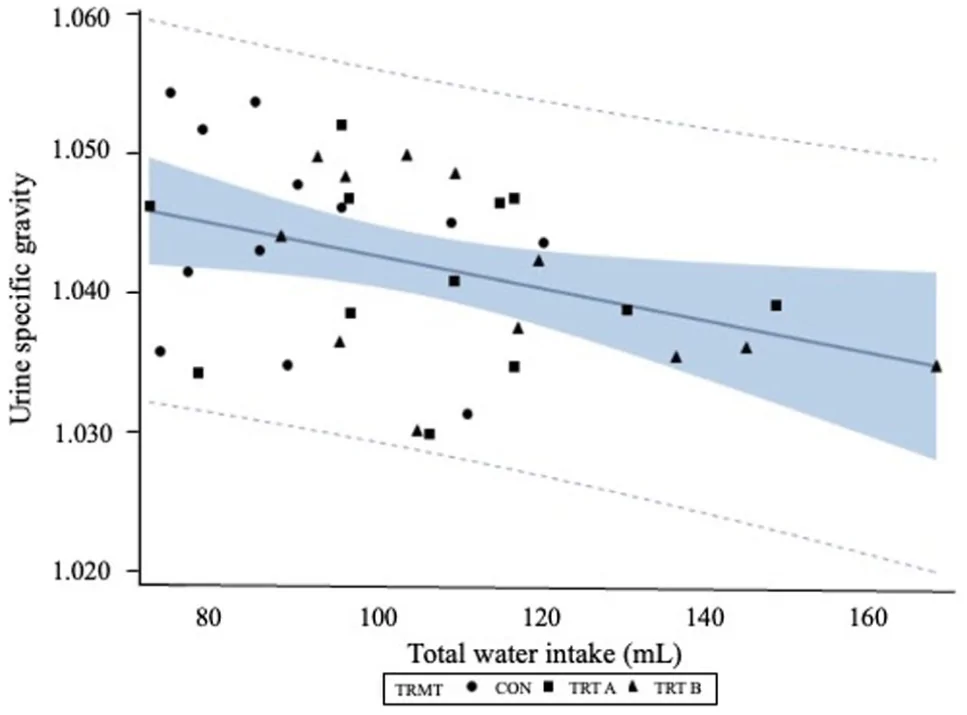
Correlation between urine specific gravity and total water intake of 12 cats provided with three dietary treatments (*P* = 0.023). The gray line represents the predicted fitted regression line (*y* = –0.00011*x* + 1.054), the blue shading represents the 95% confidence interval and the dashed lines represent the upper and lower limits. CON = dry diet; Trmt = treatment; TRT A = CON + one treat with 0.99% sodium; TRT B = CON + one treat with 1.55% sodium

A negative association between average USG and TWI was observed (*P* = 0.023; *y* = –0.00011*x* + 1.054) (Figure 1). USG decreased by an average of 0.02 and 0.03 on TRT A and TRT B when compared with the CON group, respectively (Table 4).

### Blood biochemistry

Serum biochemistry values were all within the normal physiological range for adult cats.

Serum magnesium (Mg) was greater on CON than on TRT B (*P* = 0.006), and both were similar to TRT A. No other differences were found (*P* >0.05 for all other measures) (Table 6).

**Table 6:** Serum biochemistry values of nine healthy adult male neutered cats fed a dry diet (CON), CON + one treat with 0.99% sodium (TRT A) and CON + one treat with 1.55% sodium (TRT B) for 5 days

Variable	Treatment			*P* value
	CON (n = 8)	TRT A (n = 8)	TRT B (n = 9)	
Cal mOsm (mmol/l)	313 ± 3.4	311 ± 2.3	311 ± 5.2	0.588
Lipase (U/l)	17 ± 7.9	14 ± 4.5	16 ± 9.1	0.613
Amylase (U/l)	1252 ± 237.3	1204 ± 225.4	1220 ± 219.1	0.156
CK (U/l)	119 ± 36.5	137 ± 50.0	121 ± 23.1	0.218
ALT (U/l)	41 ± 14.7	31 ± 5.4	33 ± 6.6	0.083
GGT (U/l)	0 ± 0	0 ± 0	0 ± 0	n/a
ALP (U/l)	20 ± 10.9	19 ± 7.9	18 ± 8.1	0.493
Free bilirubin (µmol/l)	1 ± 0.5	1 ± 0	1 ± 0.3	0.113
Conj bilirubin (µmol/l)	0 ± 0	0 ± 0	0 ± 0.3	1.000
Total bilirubin (µmol/l)	1 ± 0.5	1 ± 0	1 ± 0.4	0.124
Cholesterol (mmol/l)	6.57 ± 1.20	6.39 ± 1.16	6.14 ± 1.16	0.051
Glucose (mmol/l)	4.1 ± 0.3	4.3 ± 0.4	4.3 ± 0.3	0.251
Creatinine (µmol/l)	151 ± 25.8	147 ± 22.0	145 ± 23.8	0.158
Urea (mmol/l)	8.5 ± 1.3	8.5 ± 1.3	8.3 ± 1.2	0.756
Globulin (g/l)	38 ± 5.1	39 ± 5.1	38 ± 5.2	0.708
Albumin (g/l)	35 ± 2.0	35 ± 2.8	35 ± 2.3	0.753
A:G ratio	0.93 ± 0.14	0.94 ± 0.18	0.95 ± 0.15	0.894
TP (g/l)	73 ± 4.9	74 ± 4.2	73 ± 5.1	0.396
Anion (mmol/l)	28 ± 2.6	27 ± 2.3	26 ± 1.5	0.182
CO_2_ (mmol/l)	13 ± 1.3	14 ± 1.2	14 ± 1.2	0.458
Cl (mmol/l)	121 ± 1.8	120 ± 1.5	121 ± 1.9	0.627
K (mmol/l)	4.9 ± 0.3	5.0 ± 0.2	4.9 ± 0.4	0.912
Na (mmol/l)	156 ± 1.7	156 ± 1.1	156 ± 2.4	0.429
Mg (mmol/l)	1.0 ± 0.1^a^	1.0 ± 0.1^a b^	0.9 ± 0.1^b^	0.020
Na:K ratio	32 ± 2.2	32 ± 1.7	32 ± 2.9	0.842
P (mmol/l)	1.55 ± 0.10	1.63 ± 0.19	1.58 ± 0.10	0.456
Ca (mmol/l)	2.58 ± 0.06	2.56 ± 0.05	2.55 ± 0.07	0.282

Data are mean ± SD. Values within the same row with different letters are significantly different at *P* ⩽0.05

Cal mOsm/L = 1.86 ([Na] + [K]) + [glucose] + [urea]

A:G = albumin:globulin; ALP = alkaline phosphatase; ALT = alanine transaminase; Ca = calcium; Cal mOsm = calculated osmolarity; CK = creatine kinase; Cl = chlorine; CO_2_ = carbon dioxide; Conj = conjugated; GGT = gamma-glutamyl transferase; K = potassium; Mg = magnesium; Na = sodium; P = phosphorus; TP = total protein

## Discussion

High-moisture lickable treats formulated with glycine, alanine, taurine, betaine and sodium, when fed to cats, resulted in increased TWI but not FWI, and in clinical outcomes of lower USG, UP and CR but not UP:CR ratio. In previous research, when dietary moisture content was increased by providing canned,^2,17,50,53–55^ lightly cooked^3^ or dry food with added water,^15^ TWI increased while FWI decreased in colony cats. This has also been observed in privately owned cats fed 100% dry, mixed (50% wet and 50% dry) and 100% wet diets.^50,56^ In the current study, the cats maintained their drinking patterns, while the treats provided additional moisture, thereby increasing TWI without negatively affecting FWI.

In the current study, TWI was inversely correlated with USG; for every 100 ml of water consumed, USG was predicted to decrease by 0.011 (starting at a theoretical USG of 1.054 and a TWI of 0 ml). USG decreased by an average of 0.02 and 0.03 on TRT A and B, respectively. Although a direct relationship between water consumption and low USG has not previously been established in cats, similar findings have been reported. For example, cats fed a dry diet + NW had a greater daily TWI and lower USG (urine collected on days −1, 8, 15, 30 and 56) than cats fed a dry diet + tap water.^20^ The same pattern was observed in cats fed wet diets compared with those fed dry diets, as assessed using pooled q24h urine samples.^2,17^ Given the lack of consensus on the normal USG range for cats, we cannot conclude that all cats in this study were drinking enough water and were euhydrated at baseline. Importantly, cats in the CON group with the highest USG values (>1.045) showed an average decrease of 0.005 units on TRT A and 0.004 units on TRT B. This is particularly relevant given the relationship between low water intake and FLUTD^37,57,58^ and urolithiasis risk,^25,59^ and the benefit of decreasing USG in some cats.^14^ The ability of these treats to improve the hydration^60^ state in underhydrated cats and in those with FLUTD or urolithiasis warrants further investigation. However, the characterization of underhydrated, euhydrated and overhydrated as well as low-, moderate- and high-water drinkers must first be established.

Both UP and CR were lower in both treatment groups despite consistent food intake throughout the study, further suggesting that solute concentrations were diluted due to increased TWI. In a study of 534 spot-collected human urine samples, USG was positively correlated with urinary CR excretion (*r* = 0.88).^61^ However, increased TWI, resulting in decreased USG, is not observed in all studies. Cats fed a dry, soy-based diet had greater TWI than cats fed a dry, high-starch diet, but USG was not affected (urine collected q8h for 5 days).^19^ Importantly, some of the reported differences may be due to the sample collection methods or sampling periods. In humans, USG is higher when the first urine sample of the day is measured than when urine is collected later in the day;^62^ thus, results may differ between urine samples pooled over 24 h and those collected only in the morning. In the current study, urine samples were collected daily (from 0800 h to 1600 h). Importantly, changes in solute excretion or urine dilution can alter USG without influencing mOsm. These outcomes align with the physiologic meaning of these measures, whereby USG reflects the excretion of all urine solutes, including phosphates, sulphates, bicarbonates, chloride salts, ammonium salts and urea,^31^ whereas mOsm measures only circulating osmolytes. In previous studies, plasma mOsm/kg did not differ between individuals who consumed <0.74 l, <1.0 l, and <1.6 l of fluid and those who drank >2.7 l,^63^ >2.5 l,^64^ and 3.2 l^65^ of fluid, respectively; however, USG was consistently greater in the low-fluid-intake groups. This phenomenon exists because the brain constantly monitors and tightly regulates extracellular fluid concentration.

Collecting a 24- to 48-h urine sample at the end of each treatment is a common strategy for assessing USG in research settings^3,12,15,17,19,22,24,25^ or a single collection via cystocentesis in veterinary settings.^14,26^ In the current study, to maintain the group-colony dynamic and obtain daily urine samples across the 15-day study period, cats were not crated for 24-h urine collections. In a previous study with 48-h end-of-treatment urine collection periods, cats provided with NW had a lower USG, urine CR, phosphate and urine nitrogen, consistent with more dilute urine, as well as greater urine volume and TWI than cats provided with tap water.^20^ In future research, 24-h urine collections would be beneficial for measuring urine volume alongside USG and TWI to identify low- and high-water drinkers, as has been established in humans.^63–65^

The average urine pH of cats was higher in the current study than in previous reports. In earlier research, the average urine pH of cats fed a dry diet was in the range of 6.14–7.28.^9–11,19,20,24,25^ However, various environmental, physiological or methodological factors can contribute to these observed differences. Dietary urinary acidifiers, such as, but not limited to, ammonium chloride,^66,67^ methionine,^67^ phosphoric acid^11^ and sodium bisulphate,^11^ were not present in the provided diet. Although potassium chloride (KCl) was added, previous research found no difference in the urine pH of cats fed diets with or without 1.9% KCl.^23^ This effect was attributed to the overall feed base excess balance, which could not be measured in the current study. Importantly, all cats were provided with the same diet throughout the study period, and no treatment differences in urine pH were observed. Indeed, the effect of the treats provided alongside other diets, such as urinary tract diets, would be of interest. Beyond potential dietary effects, pH is sensitive to systemic acid–base responses, and fasted urine pH is lower than that of urine collected 4 and 8 h postprandially.^11^ This phenomenon is known as the alkaline tide, in which gastric hydrochloric acid is secreted for digestion, which facilitates an increase in bicarbonate, and subsequently raises blood and urinary pH.^68^ Meal size, frequency and composition can affect the alkaline tide. In a previous study, urine pH increased 2 h postprandially and remained elevated (>7.5) for 9 h in cats fed q24h; however, this was not observed in cats fed ad libitum, as larger meals empty the gastrointestinal tract more slowly than smaller meals.^66^ However, once-a-day feeding in cats can be beneficial and has been associated with improved satiety.^69^ Given that all other urinary parameters were within normal limits, the pH values in the current study validate that the collections captured the dynamic nature of urine composition.

Together, these data provide evidence that feeding either treat increased TWI and additional hydration benefits, including decreased USG, UP and CR in healthy adult male cats in a controlled environmental setting. Using a controlled colony eliminated management differences across households and allowed us to focus solely on the effect of dietary treatment. However, some limitations should be considered. To avoid overestimating the total FWI, the total FWI across the group was divided equally among all cats participating in the group drinking event and the adjusted amount was recorded. Dividing the total collective volume equally among participating cats ensured that the colony’s cumulative FWI remained accurate and prevented inflation of the overall data set. Because these group events were consistently distributed across all three experimental legs and were predominantly driven by the same cats, this variation can be better interpreted as random noise. In our mixed-model analysis, the random effect for each individual cat accounts for behavioral idiosyncrasies while still allowing accurate interpretation of the data. Finally, the study was limited to 12 neutered male cats in a controlled-environment colony, with a smaller subset of eight or nine for blood sampling. This approach provided a baseline understanding of the treatments’ impact on healthy subjects before investigating clinical cases or sex differences. Future research should examine water consumption behavior in males vs females, as well as energy balance, macronutrient oxidation, water turnover and additional urinary metabolites (eg, minerals) in healthy cats fed these hydration treats under different environmental conditions.

## Conclusions

Overall, both treats increased TWI and additional hydration benefits were reflected in decreased USG, UP and CR. No adverse effects were observed with either treatment. The variability in FWI and TWI observed under controlled environmental conditions highlights differences in cat drinking behavior across individuals. Future research should assess their effectiveness in client-owned indoor cats, where environmental and behavioral factors vary, and determine whether such treats can improve the hydration state in cats at risk of, or recovering from, underhydration, FLUTD or urolithiasis.

## Supplemental Material

Table S1 Average (mean ± SD) body weight and dry matter intake of 12 healthy adult male neutered cats fed CON, TRT A or TRT B for 5 days.
