# Supplementary material for: Consuming treats with amino acids, osmolytes and sodium increased total water intake and decreased urine specific gravity of healthy cats
Source: J Feline Med Surg. 2026 Jul 30;28(9):1098612X261477044. doi: 10.1177/1098612X261477044 (PMC13534996; doi:10.1177/1098612X261477044)
Supplement: Supplementary file 1 [file sj-docx-1-jfm-10.1177_1098612X261477044.docx]

**Supplementary Table 1.** Average (mean ± SD) body weight and dry matter intake of twelve healthy adult male neutered cats fed CON, TRT A, or TRT B for five days.

| **Variable** | **Treatment** | | | **P values** |  |  |
| --- | --- | --- | --- | --- | --- | --- |
|  | CON | TRT A | TRT B |  |  |  |
| Body weight (kg) | 4.51 ± 0.43 | 4.51 ± 0.44 | 4.53 ± 0.44 | 0.523 |  |  |
| DMI (g/d) | 50.8 ± 3.5 | 50.6 ± 4.0 | 50.6 ± 3.9 | 0.323 |  |  |

DMI = dry matter intake
